# Preterm Birth Is Associated With Immune Dysregulation Which Persists in Infants Exposed to Histologic Chorioamnionitis

**DOI:** 10.3389/fimmu.2021.722489

**Published:** 2021-08-27

**Authors:** Gemma Sullivan, Paola Galdi, Nis Borbye-Lorenzen, David Q. Stoye, Gillian J. Lamb, Margaret J. Evans, Kristin Skogstrand, Siddharthan Chandran, James P. Boardman

**Affiliations:** ^1^Medical Research Council (MRC) Centre for Reproductive Health, University of Edinburgh, Edinburgh, United Kingdom; ^2^Danish Center for Neonatal Screening, Statens Serum Institut, Copenhagen, Denmark; ^3^Department of Pathology, Royal Infirmary of Edinburgh, Edinburgh, United Kingdom; ^4^Centre for Clinical Brain Sciences, University of Edinburgh, Edinburgh, United Kingdom; ^5^Medical Research Council (MRC) Centre for Regenerative Medicine, University of Edinburgh, Edinburgh, United Kingdom

**Keywords:** complement, cytokine, fetal inflammatory response, inflammation, immunity, interleukin, preterm birth, histologic chorioamnionitis

## Abstract

**Introduction:**

Preterm infants are at increased risk of exposure to histologic chorioamnionitis (HCA) when compared to term-born controls, and this is associated with several neonatal morbidities involving brain, lungs and gut. Preterm infants could benefit from immunomodulatory therapies in the perinatal period, but development of rational treatment strategies requires improved characterization of the perinatal response to HCA. We had two objectives: The first, to characterize the umbilical cord blood immune profile in preterm infants compared to term-born controls; the second, to investigate the postnatal immune response in preterm infants exposed to HCA versus those who were not.

**Population:**

For objective one 59 term infants [mean gestational age (GA) 39^+4^ (37^+3^ to 42^+0^)] and 55 preterm infants [mean GA29^+0^(23^+3^ to 32^+0^)] with umbilical cord samples available were included; for objective two we studied 96 preterm infants [mean GA29^+1^(23^+2^ to 32^+0^)] for whom placental histology and postnatal blood samples were available.

**Methods:**

Placental histopathology was used to identify reaction patterns indicative of HCA, and a customized immunoassay of 24 inflammatory markers and trophic proteins selected to reflect the perinatal immune response was performed on umbilical cord blood in term and preterm participants and postnatal day 5 blood in the preterm group.

**Results:**

The umbilical cord blood immune profile classified gestational age category with 86% accuracy (95% CI 0.78-0.92), p-value=1.242x10^-14^. Pro-inflammatory proteins IL-6, MCP-1 and CRP were elevated in the cord blood of preterm infants whilst BDNF, C3, C9, IL-18, MMP-9 and RANTES were decreased, compared to infants born at term. In preterm infants, exposure to HCA was associated with elevations in 8 immune proteins on postnatal day 5 (BDNF, C3, C5a, C9, IL-8, MCP-1, MIP-1β and MMP-9) when compared to preterm infants who were not exposed.

**Conclusion:**

Preterm birth is associated with a distinct immune profile in umbilical cord blood and preterm infants exposed to HCA with evidence of a fetal inflammatory response have specific alterations in immune function that are apparent on day 5 of postnatal life.

## Introduction

Perinatal immune processes have a crucial role in neurodevelopment and early life inflammatory exposures are associated with an increased risk of neuropsychiatric disorders such as autism spectrum disorder, schizophrenia, bipolar disorder and depression (1, 2). Preterm infants may be exposed to multiple episodes of perinatal infection/inflammation and are particularly vulnerable to brain injury resulting from a dysregulated immune response during a critical period of CNS development (3).

Preterm infants have a distinct immune profile in umbilical cord blood and cerebrospinal fluid that includes higher levels of pro-inflammatory cytokines and lower levels of growth factors when compared to term-born controls, but there is uncertainty about the extent to which this is influenced by antenatal factors, environmental exposures and/or developmental regulation (4–6). Histologic chorioamnionitis (HCA), defined as inflammation of the chorioamniotic membranes, is strongly associated with preterm birth (7, 8) and increases the risk of neonatal morbidities including lung disease, intraventricular hemorrhage, sepsis and necrotizing enterocolitis (9–14). HCA has also been implicated in the development of white matter injury, cerebral palsy and neurodevelopmental impairment (15–18) and we have previously reported that this may be mediated by a distinct cord blood immune profile in preterm infants (19). When HCA involves a fetal inflammatory response (FIR), these risks appear to be increased further, suggesting that organ injury is mediated by a systemic fetal inflammatory response syndrome (FIRS). FIRS was initially defined using threshold values of IL-6 concentration in umbilical cord blood (20, 21), although subsequent studies have shown that histopathological FIR is associated with elevated concentrations of cytokines (IL-1β, IL-6 and TNF-α), chemokines (IL-8, MCP-1, MIP-1β, RANTES), matrix metalloproteinases (MMP-1 and MMP-9) and CRP (19, 22–26). In some preterm infants, blood concentrations of inflammatory mediators remain elevated for weeks after birth (27, 28) and may be associated with higher circulating levels of neurotrophic growth factors (29). However, neurotrophic capability following exposure to intrauterine inflammation is not well understood and previous study designs leave uncertainty about the role of the complement system in perinatal inflammation, which plays a critical role in the innate immune response.

Preterm infants could benefit from immunomodulatory therapies in the perinatal period, but development of rational treatment strategies requires improved characterization of the neonatal immune profile and the postnatal response to HCA. In this study, an immunoassay of 24 analytes customized to reflect the perinatal immune response was used to analyze profiles from umbilical cord and postnatal blood with placental histopathology to (1) characterize the intrauterine immune environment of preterm infants compared to term-born controls, and (2) test the hypothesis that exposure to histologic chorioamnionitis is associated with an altered immune and neurotrophic profile in the first week after very preterm birth.

## Materials and Methods

### Study Population

Term (GA>37 weeks) and preterm (GA<33 weeks) infants were recruited to a longitudinal cohort study designed to investigate the effect of preterm birth on brain development, at the Royal Infirmary of Edinburgh, UK (30). Ethical approval was obtained from the UK National Research Ethics Service and parents provided written informed consent (South East Scotland Research Ethics Committee 16/SS/0154). For objective 1, we included infants if umbilical cord blood samples were available (59 preterm and 55 term), and for objective 2 we included preterm infants with placental histopathology and postnatal blood samples (n=96).

### Dried Blood Spot Sample Analysis

Dried blood spot samples (DBSS) were taken from the umbilical cord following delivery for both preterm cases and term-born controls. For preterm infants, an additional sample was collected on day 5 of life. A customized multiple sandwich immunoassay based on Meso-Scale technology was used to measure blood spot levels of Interleukin(IL)1-β, IL-2, IL-4, IL-5, IL-6, IL-8, IL-10, IL-12p70, IL-17, IL-18, Monocyte chemotactic protein-1 (MCP-1), Macrophage inflammatory protein-1α (MIP-1α), Macrophage inflammatory protein-1β (MIP-1β), Tumor necrosis factor-α (TNF-α), Tumor necrosis factor-β (TNF-β), Brain-derived neurotropic factor (BDNF), Granulocyte-macrophage colony-stimulating factor (GM-CSF), Interferon-γ (IFN-γ), C-reactive protein (CRP), matrix-metalloproteinase 9 (MMP-9), Regulated upon activation, normal T cell expressed and secreted (RANTES) and Complement components C3, C5a and C9.

Two 3.2 mm disks from the DBSS were punched into each well of Nunc 96-well polystyrene microwell plates (#277143, Thermo Fisher Scientific). 130 µl extraction buffer (PBS containing 1% BSA (Sigma Aldrich #A4503), 0.5% Tween-20 (#8.22184.0500, Merck Millipore), and complete protease inhibitor cocktail (#11836145001, Roche Diagnostics) were added to each well, and the samples were incubated for 1 hour at room temperature on a microwell shaker set at 900 rpm. The extracts were analysed using U-plex plates (Meso-Scale Diagnostics (MSD), Maryland, US) coated with antibodies specific for IL-1β, IL-2, IL-4, IL-5, IL-6, IL-8, IL-12, IL-17, TNF-α, MIP-1β on one plate (#K15067 customized) and BDNF, GM-CSF, IL-10, IL-18, IFN-γ, TNF-β, MCP-1, MIP-1α on another (#K151AC customized) (both MSD). Supplier’s instructions were followed, and extracts were analysed undiluted. A third multiplex analysis was developed in-house applying extracts diluted 1:10 in diluent 7 (#R54BB, MSD) using antibodies specific for C3 (HYB030-07 and HYB030-06, SSI Antibodies, Copenhagen, Denmark), C5a (10604-MM04 and 10604-MM06, Sino Biological, Eschborn, Germany), C9 (R-plex kit #F21XZ, MSD), MMP-9 (BAF911 and MAB911), RANTES (MAB278 and AF278NA) and CRP (BAM17072 and MAB1701) (all R&D Systems, Minneapolis, US) for coating the U-plex plate and for detection, respectively. Coating antibodies (used at 1 µg/mL, except CRP used at 10 ng/mL) were biotinylated (using EZ-Link Sulfo-NHS-LC-Biotin #21327, Thermo Fisher Scientific) in-house (if not already biotinylated at purchase) and detection antibodies were SULFO-tagged (R91AO, MSD), both at a challenge ratio of 20:1. The following calibrators were used: C3: #PSP-109 (Nordic Biosite, Copenhagen, DK), C5a: #10604-HNAE (Sino Biological), C9: #F21XZ (from R-plex kit, MSD), MMP-9: #911-MP, RANTES: #278-RN and CRP: #1707-CR/CF (all from R&D Systems). Calibrators were diluted in diluent 7, detection antibodies (used at 1 µg/mL, except CRP used at 100 ng/mL) were diluted in diluent 3 (#R50AP, MSD). Controls were made in-house from part of the calibrator solution in one batch, aliquoted in portions for each plate and stored at -20°C until use. The samples were prepared on the plates as recommended from the manufacturer and were immediately read on the QuickPlex SQ 120 (MSD). Analyte concentrations were calculated from the calibrator curves on each plate using 4PL logistic regression using the MSD Workbench software. Intra-assay variations were calculated from 16 measurements of a pool of the same control sample on the same plate. Inter-assay variations were calculated from controls analysed in duplicate on each plate during the sample analysis, 4 plates in total. Limits of detection were calculated as 2.5 standard deviations from duplicate measurements of the zero calibrator. The higher detection limit was defined as the highest calibrator concentration. Median intra-assay variation was 8.2% and median inter-assay variation was 11.1%. Detection limits are detailed in **Table S1**.

### Placental Examination

Placental examination was performed by an experienced perinatal pathologist (M.J.E.) and placental reaction patterns were reported according to the site of inflammation, using a structured system (31). HCA was defined as the presence of an inflammatory response in the placental membranes of any grade or stage. Maternal inflammatory response (MIR) was defined as the presence of chorionitis, chorioamnionitis or intervillositis. Fetal inflammatory response (FIR) was defined as the presence of vasculitis in the chorionic plate or funisitis involving any vessel of the umbilical cord.

### Statistical Analysis

Participant demographics are described as mean ± SD if normally distributed and mean (range) if skewed. Student’s T test or Mann-Whitney U were used to compare distributions, and Chi-square tests were used to compare proportions. Analytes with values less than the level of detection (<LOD) were assigned the lowest detectable level prior to statistical analysis, and analytes with concentrations <LOD in ≥75% of samples were excluded from subsequent statistical analysis. Data normality testing with Shapiro-Wilk confirmed a non-normal distribution of analyte concentrations, so to investigate group differences in blood immune mediator profiles we used the Mann-Whitney U or Kruskal Wallis and post hoc Dunn test, with Bonferroni correction for multiple tests.

Principal component analysis (PCA) was used to identify analytes contributing to variance in the cord blood profile and analytes that contributed to PCs with eigenvalues >1 were then entered as independent variables in a logistic regression model to predict preterm or term category. Analytes contributing to variability within PCs predictive of gestational category were then investigated individually using Spearman’s rank order correlation to identify developmentally regulated analytes most strongly correlated with gestational age. Statistical analyses were performed using SPSS version 24.0 (IBM Corp., Armonk, NY), with the exception of PCA, which was performed using R version 3.6.1 (R Core Team, 2019).

## Results

### Umbilical Cord Blood Profile Associated With Preterm Birth

**Figure 1:**
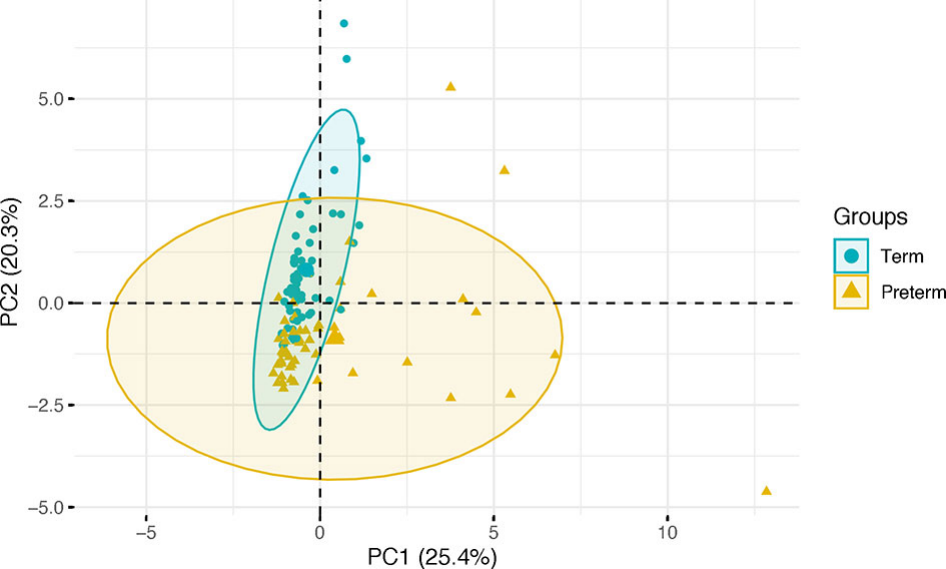
Projection of individual cord blood inflammatory profiles onto the first two principal components, grouped by gestational age category. Term infants are represented in green and preterm infants in gold, with ellipses modelled on the mean and covariances of each group.

Umbilical cord blood samples were available for 59 term and 55 preterm infants. Participant characteristics are detailed in **Table 1**. 10 analytes (GM-CSF, IFN-γ, IL-2, IL-4, IL-5, IL-10, IL-12, IL-17, MIP-1α and TNF-β) were <LOD in ≥75% of samples and were therefore excluded from subsequent analysis. Median and interquartile range of analytes are shown in **Table 2**. There were significant group differences for 9 immune mediators (p<0.004, Bonferroni corrected). Pro-inflammatory proteins IL-6, MCP-1 and CRP were elevated in the cord blood of preterm infants whilst BDNF, C3, C9, IL-18, MMP-9 and RANTES were decreased compared to controls born at term. PCA showed that five principal components (eigenvalues>1) explained 76% of the variance in the cord blood profile with the majority of variance explained by the first two components (25% and 20% respectively, **Table S2**). Projection of individual inflammatory profiles onto the first two principal components is shown in **Figure 1**.

**Table 1:** Clinical characteristics of participants for umbilical cord blood analysis.

	Preterm n = 55	Term n = 59	p-value
Mean gestational age, weeks (range)	29^+0^ (23^+3^-32^+0^)	39^+4^ (37^+3^-42^+0^)	<0.001
Mean birthweight, g (range)	1202 (454-2110)	3549 (2556-4800)	<0.001
Mean birthweight z-score (SD)	-0.2696 (1.24)	0.6828 (1.08)	<0.001
Male sex, n (%)	33 (60)	30 (51)	0.326
Maternal factors			
BMI, mean (SD)	26.2 (5.8)	26.4 (5.5)	0.845
Pre-eclampsia, n (%)	3 (5)	3 (5)	0.930
Antenatal steroids, n (%)	54 (98)	NA	NA
Antenatal magnesium sulphate, n (%)	52 (95)	NA	NA
Delivery mode, n (%):			
Vaginal	23 (42)	23 (39)	0.484
Caesarean	32 (58)	36 (61)	0.223
-Pre-labor	22 (40)	27 (46)	0.506
-In labor	10 (18)	9 (15)	
Any labor, n (%)	33 (60)	32 (54)	0.535
Histologic chorioamnionitis, n (%)	24 (44)	10 (17)	0.002
MIR+ FIR-	11	2	
MIR+ FIR+	13	8	

NA, Not applicable.

**Table 2:** Cord blood analytes in preterm infants and term-born controls.

Analyte (pg/ml)	Preterm n = 55		Term n = 59		p-value*
	Median	Q1, Q3	Median	Q1, Q3	
BDNF	22.27	6.04, 36.90	62.33	43.18, 113.68	<0.001
C3	3081995.73	1858060.85, 4569905.86	4447458.83	3520957.75, 5613382.51	<0.001
C5a	3694.52	2420.56, 11470.85	7136.48	4059.83, 9443.64	0.004
C9	1236.73	215.00, 6901.99	12628.69	3608.60, 43156.07	<0.001
CRP	100.88	89.00, 12054.79	89.00	89.00, 89.00	<0.001
IL-1β	0.26	0.26, 0.49	0.11	0.02, 0.34	0.016
IL-6**	0.45	0.45, 6.00	0.45	0.45, 0.45	<0.001
IL-8	12.05	5.75, 81.08	8.29	4.89, 14.71	0.134
IL-18	25.62	10.35, 38.34	41.27	27.50, 54.78	0.003
MCP-1	109.09	68.69, 210.75	48.12	38.54, 66.63	<0.001
MIP-1β	12.01	7.47, 18.82	9.72	6.88, 18.24	0.331
MMP-9	14000.68	4485.50, 40077.02	155885.89	87194.67, 358084.73	<0.001
RANTES	1460.12	702.74, 2754.37	3127.55	1817.10, 5295.39	<0.001
TNF-α	0.27	0.27, 0.37	0.27	0.27, 0.27	0.304

*Mann Whitney U test to compare ranks (Bonferroni corrected threshold p < 0.004). **IL-6: For preterm infants: 27 were below the lower limit of detection (0.45pg/ml), and the range for the remaining 28 was 0.46-249.56pg/ml. For term infants: 50 were below the lower limit of detection and the range for the remaining 9 was 0.66-11.46pg/ml.

**Figure 2:**
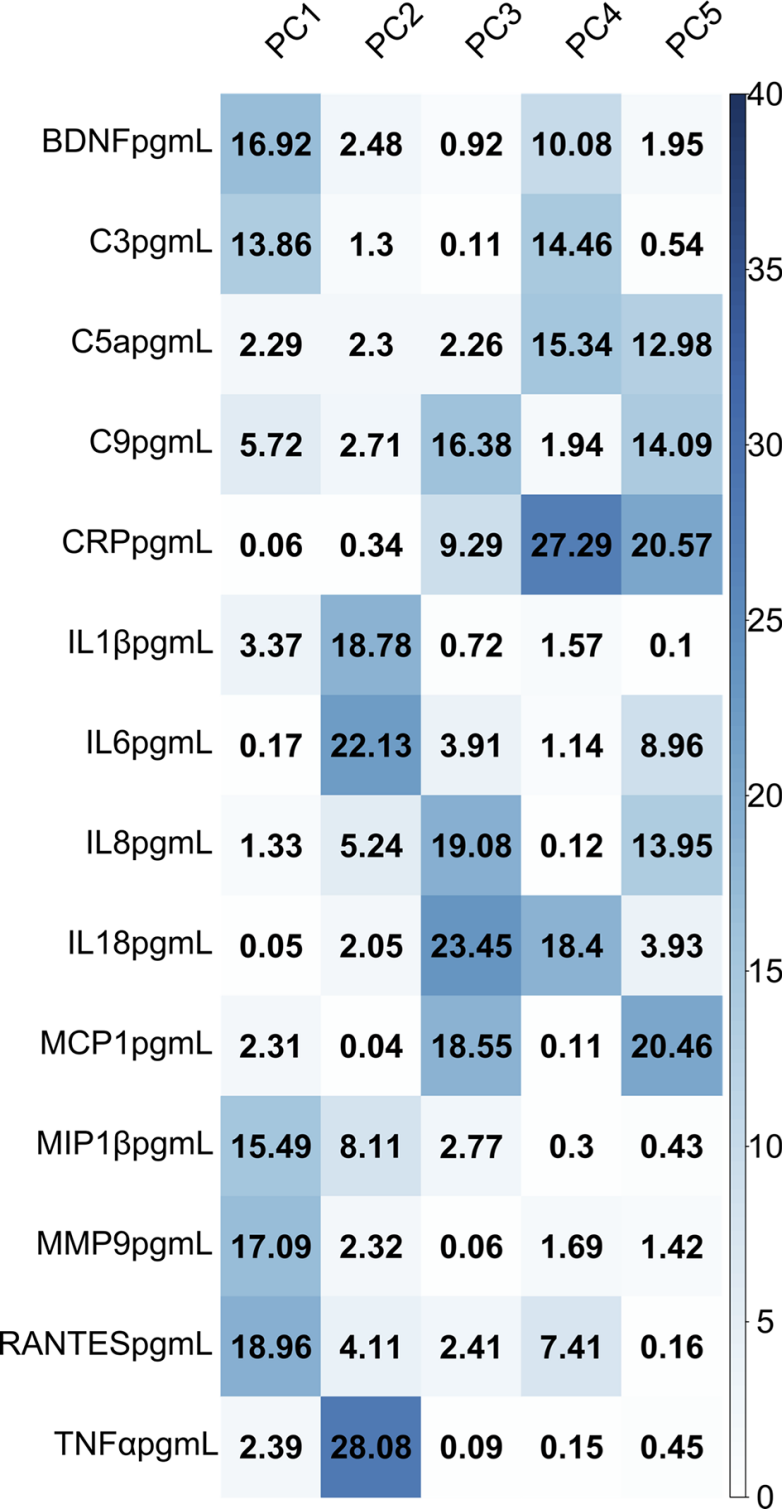
Heatmap demonstrating the percentage contribution of each analyte to variability in the cord blood profile, grouped by principal component.

In a logistic regression model to predict preterm or term category based on cord blood profile, principal components predicted gestational age category with a classification accuracy of 86% (95% CI 0.78-0.92), p value= 1.242x10^-14^ (**Table 3**). The percentage contribution of each analyte to the principal components is shown in **Figure 2**. Amongst immune mediators contributing to variability within the principal components that predicted gestational category, correlation analysis showed that cord blood MMP-9 and BDNF were highly correlated with gestational age at birth (rho >0.65, p<0.001) (**Table 4**).

**Table 3:** Logistic regression for the prediction of gestational age category using principal components derived from the umbilical cord blood profile.

	B	β	p-value
PC1	2.8189	5.3424	0.000337
PC2	-2.6162	-4.4331	1.71e-06
PC3	-0.1343	-0.1850	0.648519
PC4	0.8840	1.0084	0.135172
PC5	-0.8628	-0.9076	0.027207

**Table 4:** Correlation between individual analytes that contributed to the principal components predictive of gestational category and gestational age at birth.

Analyte	Spearman’s rho	p-value
MMP-9	0.685	4.1162x10^-17^
BDNF	0.654	2.8795x10^-15^
RANTES	0.346	0.000160
C3	0.290	0.002
IL-1β	0.190	0.043

### Histologic Chorioamnionitis Is Associated With an Altered Immune Profile on Day 5 After Very Preterm Birth

Ninety-six preterm infants had placental histopathology and postnatal day 5 blood samples. Participant characteristics are detailed in **Table 5**. Thirty-one infants (32%) were exposed to HCA and 18 of those had histopathological evidence of a fetal inflammatory response. Infants with HCA exposure had lower GA at birth than infants without HCA: mean GA 28^+2^ weeks versus 29^+4^ weeks (p=0.03), were more likely to have been delivered vaginally (p<0.001) and more likely to have prolonged rupture of membranes prior to delivery (p<0.001). However, there were no statistically significant group differences in exposure to antenatal steroids or magnesium sulphate, birthweight or infant sex.

**Table 5:** Characteristics of preterm infants with day 5 blood samples.

	No HCA n = 65	HCA n = 31	p-value
Mean gestational age, weeks (range)	29^+4^ (24^+0^-32^+0^)	28^+2^ (23^+2^-32^+0^)	0.030
Mean birthweight, g (range)	1246 (454-1915)	1187 (500-2060)	0.447
Mean birthweight z-score (SD)	-0.3287 (1.23)	0.1870 (0.69)	0.010
Male sex, n (%)	37 (57)	18 (58)	0.916
Antenatal steroids, n (%)	61 (94)	30 (97)	0.546
Antenatal magnesium sulphate, n (%)	59 (91)	30 (97)	0.290
Delivery mode, n (%):			
Vaginal	15 (23)	23 (74)	<0.001
Caesarean	50 (77)	8 (26)	0.003
-Pre-labor	34 (68)	8 (100)	0.033
-In labor	16 (32)	0 (0)	
Any labor, n (%)	31 (48)	23 (74)	0.014
Prolonged rupture of membranes, n (%)	8 (12)	14 (45)	<0.001
Early onset sepsis, n (%)	4 (6)	4 (13)	0.263
Late onset sepsis, n (%)	9 (14)	4 (13)	0.955
Bronchopulmonary dysplasia, n (%)	18 (28)	12 (39)	0.189
Necrotizing enterocolitis, n (%)	3 (5)	5 (16)	0.042
Retinopathy of prematurity, n (%)	3 (5)	5 (16)	0.042

Prolonged rupture of membranes: >24 hours before delivery. Sepsis: Positive blood culture with a pathogenic organism and/or antibiotic treatment course for ≥5 days. Early-onset sepsis: <72 hours after birth, late-onset sepsis: >72 hours after birth. Bronchopulmonary dysplasia: supplemental oxygen therapy or respiratory support at 36 + ^0^ weeks gestational age. Necrotizing enterocolitis: medical treatment for ≥7 days or surgical treatment. Retinopathy of prematurity: requiring treatment.

The concentrations of eight analytes (BDNF, C3, C5a, C9, IL-8, MCP-1, MIP-1β and MMP-9) differed on day 5 in preterm infants who were exposed to HCA compared to those who were not exposed (Mann Whitney U test, p<0.05). These differences appear to be driven by histological evidence of a fetal inflammatory response for all but C5a (Kruskal Wallis with Dunn’s *post hoc* test, p<0.05) (**Table 6**).

**Table 6:** Day 5 blood analyte concentrations for preterm infants exposed to histologic chorioamnionitis compared to those not exposed.

Analyte (pg/ml)	No HCA (65)		HCA		HCA		p-value*
			MIR+ FIR- (13)		MIR+ FIR+ (18)		
	Median	Q1, Q3	Median	Q1, Q3	Median	Q1, Q3	
**BDNF**	**28.49**	**14.27, 42.74**	**35.44**	**18.60, 75.22**	**44.19**	**27.99, 111.02**	**0.024**
**C3**	**3584453.76**	**2761102.54, 4875046.42**	**4180179.50**	**3123653.67, 4837608.71**	**6554839.71**	**4922502.77, 7576252.57**	**<0.001**
C5a	6457.25	4218.14, 9987.78	10511.70	7785.16, 13840.42	9145.47	7178.41, 13079.57	0.004
**C9**	**7462.57**	**2455.08, 20710.16**	**12407.86**	**2922.06, 58688.26**	**26040.08**	**7899.24, 63062.60**	**0.042**
CRP	248.98	89.00, 8104.18	467.95	89.00, 7119.96	5306.99	89.00, 68707.42	0.156
IL-1β	0.03	0.03, 0.08	0.03	0.03, 0.07	0.07	0.03, 0.26	0.058
IL-6	0.45	0.45, 0.45	0.45	0.45, 0.45	0.45	0.45, 0.45	0.870
**IL-8**	**11.44**	**7.61, 19.30**	**19.85**	**5.97, 35.31**	**30.61**	**19.91, 44.26**	**0.008**
IL-18	35.78	21.10, 53.64	37.80	20.21, 62.48	30.63	22.69, 45.71	0.463
**MCP-1**	**135.02**	**108.41, 212.56**	**115.70**	**98.02, 222.52**	**97.80**	**77.12, 140.04**	**0.012**
**MIP-1β**	**7.88**	**5.50, 10.95**	**9.14**	**5.94, 14.61**	**12.61**	**8.33, 22.97**	**0.015**
**MMP-9**	**56654.38**	**28317.00, 99300.57**	**152090.56**	**44718.53, 300102.68**	**345833.11**	**151784.29, 947473.72**	**<0.001**
RANTES	2606.92	910.54, 4199.97	3079.02	1830.27, 4439.981	3970.04	2256.12, 5932.24	0.141
TNF-α	0.27	0.27, 0.27	0.27	0.27, 0.27	0.27	0.27, 0.59	0.502

*Kruskal-Wallis test comparing three groups. Analytes highlighted in bold demonstrated significant group differences in a pairwise comparison of no HCA versus MIR+ FIR+ groups using post hoc Dunn’s test. For C5a, post hoc tests showed the contrast was due to no HCA versus MIR+ FIR-.

## Discussion

By combining placental histopathology with a customized array of immune mediators in umbilical cord blood and postnatal blood from a large group of mother-infant dyads, this study characterizes differences in the systemic immune profile of very preterm infants compared with term-born controls, and demonstrates that exposure to HCA with evidence of a fetal inflammatory response is associated with postnatal immune dysregulation on day 5 after birth.

The umbilical cord blood immune profile was distinctly pro-inflammatory in preterm infants with significant elevations in proteins associated with the acute phase response: IL-6, MCP-1 and CRP. In contrast, six proteins were elevated in healthy term-born controls, suggesting developmental regulation with increasing gestational age: BDNF, C3, C9, IL-18, MMP-9 and RANTES. Eight proteins were increased on postnatal day 5 in preterm infants exposed to HCA compared to preterm infants without HCA: BDNF, C3, C5a, C9, IL-8, MCP-1, MIP-1β and MMP-9. Our findings are consistent with previous studies showing that the neonatal systemic inflammatory response can be dysregulated and prolonged in the weeks after preterm birth (4, 27, 28, 32), but additionally suggest that this is programmed by a fetal inflammatory response.

BDNF expression has previously been shown to correlate with gestational age and postnatal age (29, 33) but here we show upregulation in preterm infants exposed to HCA with evidence of a fetal inflammatory response. BDNF belongs to the family of neurotrophins: an important group of signaling molecules responsible for neuronal growth, maturation and synaptic plasticity during development (34). Prematurity, placental dysfunction and fetal growth restriction have all been associated with reduced levels of BDNF (29, 35–37) which may have important implications for long-term brain health. Reduced BDNF in the neonatal period has been associated with increased risk of developing autism spectrum disorder (38) whilst elevated BDNF in the weeks after preterm birth is associated with better cognitive performance in childhood (39, 40).

C3 was identified as a novel postnatal marker of exposure to intrauterine inflammation. The complement cascade plays a key role in the innate immune response (41) but is a potent inflammatory system which when dysregulated can cause significant tissue damage following injury. The complement cascade can be activated through several mechanisms, but all component pathways converge at Complement protein C3 (42). C3 participates in multiple key processes affecting developing brain architecture, including tagging of synapses for pruning by microglia (43, 44). The complement system is under-developed in preterm infants and complement regulators are low (45, 46), which may contribute to an uncontrolled complement response in the context of inflammation (47). We have previously shown that the downstream anaphylatoxin, C5a is elevated in the cerebrospinal fluid (CSF) of preterm infants when compared to term-born controls (6) and numerous studies beyond the neonatal period have also implicated complement C3 dysregulation in CNS pathology including neurodevelopmental disorders (48), multiple sclerosis (49), traumatic brain injury (50) and neurodegeneration (51).

MMP-9 on postnatal day 7 has previously been shown to correlate with the severity of funisitis following extremely preterm birth (27). MMP-9 is a member of the zinc-dependent endopeptidases that prototypically cleave extracellular matrix (ECM), cell adhesion molecules and cell surface receptors. Matrix-metalloproteinases also modulate the inflammatory response through the regulation of endothelial barrier function, cytokine activity and chemotactic gradient formation (52). The ECM is a key regulator of neural network development and plasticity through the stabilization of synaptic contacts. Dysregulation of MMP-9 during a critical window of CNS vulnerability may therefore have long-term consequences on structural connectivity (53). MMP-9 is higher in the CSF of preterm infants when compared to term-born controls and also higher amongst preterm infants with post hemorrhagic ventricular dilatation (PHVD) when compared to those without brain injury (6, 54). Elevated plasma MMP-9 is also associated with hypoxic-ischemic encephalopathy, correlating with severity of injury in human infants born at term (55–57).

The data provide new insights into immune dysregulation in the context of HCA and support several lines of evidence that suggest a link between HCA, immune response and neurodevelopment. First, activation of the fetal inflammatory response is an independent predictor of neonatal morbidity after adjustment for gestational age and obstetric indication for delivery (20). Second, sequencing studies have shown that exposure to HCA results in fetal immune programming in preterm infants with modulation of monocyte responses (58–60). Third, intra-amniotic inflammation is associated with adverse perinatal outcomes whether or not microbes are detected (61). Fourth, exposure to HCA has been associated with an increased risk of intraventricular hemorrhage (62), white matter injury (17, 18) and neurodevelopmental impairment (15, 63) in children who were delivered preterm. Finally, intrauterine infection or stimulation with lipopolysaccharide can induce a systemic fetal inflammatory response, neuroinflammation and white matter injury (64–66). It is estimated that 3-5% of infants born by spontaneous vaginal delivery at term are exposed to HCA (67). Given that HCA is associated with cerebral palsy among children born at term (68), and maternal immune activation in term pregnancies is linked to neurodevelopmental and psychiatric diagnoses (69), further work is warranted to determine the relevance of our observations in preterm infants to births at term complicated by HCA.

Strengths of the study include investigation of a large number of inflammation-associated proteins representative of the perinatal immune response, and a data driven approach to characterize the inflammatory profile associated with very preterm birth and exposure to HCA. A limitation of the study is that the concentration of anti-inflammatory cytokines IL-4 and IL-10 were below the level of detection in our participants and so inferences about the balance of damaging and protective factors could not be explored. Another limitation is that amniotic fluid was not available for microbial analysis. Recent transcriptomic studies have shown that the presence of a fetal inflammatory response is more strongly associated with microbial invasion rather than sterile inflammation (70) but an alternative study design would be required to investigate differences in the postnatal immune profile of infants exposed to intra-amniotic infection compared to sterile inflammation.

Our data suggest that systemic fetal inflammation modulates neurotrophic capability and complement system activation in the perinatal period. However, a larger sample size would be required to perform sub-group analyses based on gestational age or sex, and to investigate possible confounding by antenatal steroids, mode of delivery and postnatal events.

Future work with a larger sample size and a replication cohort is needed to investigate the relationship between immune profiles and lung disease, gastrointestinal complications and neurocognitive outcomes following preterm birth, and to test for causality.

By combining placental histopathology with a comprehensive assessment of the immune response, we have shown that very preterm infants have a distinct pro-inflammatory profile in umbilical cord blood and that fetal inflammation is associated with an altered neonatal immune profile on postnatal day 5. These results focus research attention on improved detection of fetuses exposed to intrauterine inflammation and suggest there may be a therapeutic window for targeted intervention that could reduce the risk of co-morbidities associated with HCA.

## Data Availability Statement

The raw data supporting the conclusions of this article will be made available by the authors, without undue reservation.

## Ethics Statement

Ethical approval was obtained from the UK National Research Ethics Service and parents provided written informed consent (South East Scotland Research Ethics Committee 16/SS/0154).

## Author Contributions

GS conceived and designed the study, acquired and analyzed data, and drafted the article. PG and ME analyzed data and revised the article critically for important intellectual content. NB-L and KS acquired data, analysed data and revised the article critically for important intellectual content. DS and GL acquired data, and revised the article critically for important intellectual content. SC supervised acquisition of data and revised the article critically for important intellectual content. JB conceived and designed the study, supervised acquisition of data, analysed data and drafted the article. All authors contributed to the article and approved the submitted version.

## Funding

Financial support for this study was provided by Theirworld (www.theirworld.org). This work was undertaken in the Medical Research Council Centre for Reproductive Health, which is funded by a Medical Research Council Centre grant (Medical Research Council G1002033).

## Conflict of Interest

The authors declare that the research was conducted in the absence of any commercial or financial relationships that could be construed as a potential conflict of interest.

## Publisher’s Note

All claims expressed in this article are solely those of the authors and do not necessarily represent those of their affiliated organizations, or those of the publisher, the editors and the reviewers. Any product that may be evaluated in this article, or claim that may be made by its manufacturer, is not guaranteed or endorsed by the publisher.
