# Supplementary material for: Preterm Birth Is Associated With Immune Dysregulation Which Persists in Infants Exposed to Histologic Chorioamnionitis
Source: Front Immunol. 2021 Aug 27;12:722489. doi: 10.3389/fimmu.2021.722489 (PMC8430209; doi:10.3389/fimmu.2021.722489)
Supplement: Supplementary file 1 [file DataSheet_1.docx]

Supplementary Material

# Supplementary Tables

**Table S1. Dried blood spot sample detection limits and assay variations.**

| **Analyte** | **Lower detection limit (pg/mL)** | **Higher detection limit (pg/mL)** | **Intra-assay CV%** | **Inter-assay CV%** |
| --- | --- | --- | --- | --- |
| BDNF | 1.05 | 41000 | 2.71 | 3.84 |
| C3 | 13739 | 50000000 | 3.62 | 15.20 |
| C5a | 544 | 16700000 | 5.60 | 19.10 |
| C9 | 8.38 | 3300000 | 5.60 | 12.80 |
| CRP | 89.0 | 100000000 | 16.70 | 34.50 |
| GM-CSF | 0.101 | 12500 | 4.19 | 7.86 |
| IFN-γ | 0.646 | 34875 | 3.24 | 6.29 |
| IL-1β | 0.0260 | 4788 | 11.90 | 10.80 |
| IL-2 | 0.340 | 2688 | 11.30 | 14.70 |
| IL-4 | 0.00956 | 2700 | 16.10 | 16.80 |
| IL-5 | 0.125 | 5125 | 12.00 | 9.67 |
| IL-6 | 0.452 | 2650 | 11.10 | 20.9 |
| IL-8 | 0.0684 | 2575 | 10.9 | 15.0 |
| IL-10 | 0.0886 | 4625 | 4.13 | 5.56 |
| IL-12p70 | 0.157 | 9063 | 10.30 | 17.00 |
| IL-17 | 0.286 | 47375 | 13.50 | 11.40 |
| IL-18 | 0.199 | 50500 | 3.36 | 6.58 |
| MCP-1 | 1.28 | 7400 | 2.55 | 6.99 |
| MIP-1α | 0.960 | 7500 | 2.71 | 6.32 |
| MIP-1β | 1.73 | 2325 | 13.8 | 5.39 |
| MMP-9 | 24.1 | 5000000 | 6.29 | 25.10 |
| RANTES | 37.9 | 1600000 | 10.10 | 29.00 |
| TNF-α | 0.272 | 4538 | 11.80 | 4.48 |
| TNF-β | 0.0250 | 5050 | 2.52 | 3.90 |

**Table S2. Variance in the cord blood inflammatory profile.**

| **Component** | **Eigenvalue** | **Total variance %** | **Cumulative variance %** |
| --- | --- | --- | --- |
| 1 | 3.56 | 25.43 | 25.43 |
| 2 | 2.85 | 20.33 | 45.76 |
| 3 | 1.88 | 13.44 | 59.20 |
| 4 | 1.29 | 9.21 | 68.42 |
| 5 | 1.10 | 7.84 | 76.25 |
| 6 | 0.85 | 6.07 | 82.32 |
| 7 | 0.58 | 4.13 | 86.45 |
| 8 | 0.50 | 3.55 | 90.00 |
| 9 | 0.46 | 3.32 | 93.31 |
| 10 | 0.31 | 2.22 | 95.53 |
| 11 | 0.20 | 1.45 | 96.98 |
| 12 | 0.19 | 1.38 | 98.36 |
| 13 | 0.18 | 1.31 | 99.67 |
| 14 | 0.05 | 0.33 | 100.00 |
